# Enhancing unmanned ground vehicle performance in SAR operations: integrated gesture-control and deep learning framework for optimised victim detection

**DOI:** 10.3389/frobt.2024.1356345

**Published:** 2024-06-18

**Authors:** Muhammad Hamza Zafar, Syed Kumayl Raza Moosavi, Filippo Sanfilippo

**Affiliations:** ^1^ Department of Engineering Sciences, University of Agder, Grimstad, Norway; ^2^ Department of Software Engineering, Kaunas University of Technology, Kaunas, Lithuania

**Keywords:** disaster response, unmanned ground vehicles (UGVs), deep learning, multi-modal fusion, human detection

## Abstract

In this study, we address the critical need for enhanced situational awareness and victim detection capabilities in Search and Rescue (SAR) operations amidst disasters. Traditional unmanned ground vehicles (UGVs) often struggle in such chaotic environments due to their limited manoeuvrability and the challenge of distinguishing victims from debris. Recognising these gaps, our research introduces a novel technological framework that integrates advanced gesture-recognition with cutting-edge deep learning for camera-based victim identification, specifically designed to empower UGVs in disaster scenarios. At the core of our methodology is the development and implementation of the Meerkat Optimization Algorithm—Stacked Convolutional Neural Network—Bi—Long Short Term Memory—Gated Recurrent Unit (MOA-SConv-Bi-LSTM-GRU) model, which sets a new benchmark for hand gesture detection with its remarkable performance metrics: accuracy, precision, recall, and F1-score all approximately 0.9866. This model enables intuitive, real-time control of UGVs through hand gestures, allowing for precise navigation in confined and obstacle-ridden spaces, which is vital for effective SAR operations. Furthermore, we leverage the capabilities of the latest YOLOv8 deep learning model, trained on specialised datasets to accurately detect human victims under a wide range of challenging conditions, such as varying occlusions, lighting, and perspectives. Our comprehensive testing in simulated emergency scenarios validates the effectiveness of our integrated approach. The system demonstrated exceptional proficiency in navigating through obstructions and rapidly locating victims, even in environments with visual impairments like smoke, clutter, and poor lighting. Our study not only highlights the critical gaps in current SAR response capabilities but also offers a pioneering solution through a synergistic blend of gesture-based control, deep learning, and purpose-built robotics. The key findings underscore the potential of our integrated technological framework to significantly enhance UGV performance in disaster scenarios, thereby optimising life-saving outcomes when time is of the essence. This research paves the way for future advancements in SAR technology, with the promise of more efficient and reliable rescue operations in the face of disaster.

## 1 Introduction

In contemporary robotics, the control interface plays a pivotal role in realising the full potential of these machines, especially in contexts as critical as search and rescue (SAR) operations. Hand gesture-based control emerges as a highly relevant and promising avenue, recognising the intrinsic connection between human communication and motion. Traditional input methods, such as joysticks or keyboards (Usakli and Gurkan, 2009), often impose a steep learning curve and can be cumbersome in dynamic and high-stress situations. In contrast, harnessing hand gestures as a control modality not only aligns with natural human movements but also offers an intuitive and immediate way to direct robotic actions. This fusion of human-centric communication and robotic dexterity holds immense potential to revolutionise SAR efforts, optimising coordination and response times in lifesaving scenarios.

When considering alternative approaches, speech recognition stands as a highly convenient communication method. However, it faces challenges due to the diverse range of human accents and struggles in noisy, uncontrollable environments. On the other hand, vision-based approaches utilising facial expressions, eye tracking, or head movements have been explored Castillo et al. (2018). Among these, gesture recognition is the most comprehended. Vision-based Human-Robot Interaction (HRI) technology offers a non-contact approach capable of conveying intricate information effectively Chen et al. (2019). Among all human body gestures, the hands are a natural focal point, given their intuitive use in human-to-human communication. The primary objective of pursuing touch-less methods is to foster engagement between robots and humans, ultimately facilitating a natural interaction. Although hand gesture recognition is an area of extensive study Rautaray and Agrawal (2015) it still encounters challenges such as complex and dynamic backgrounds or varying illumination conditions Gao et al. (2017). In this study, our focus lies on recognising hand gestures as a means of easy and natural communication with robots. This recognition is vital for robotics applications such as HRI or assisted robotics, aiming for a seamless, effective interaction with minimal intrusion Wachs et al. (2011).

HRI stands at the forefront of this revolution, especially in SAR missions where seamless interaction and collaboration between humans and robots are paramount. HRI technologies enable effective coordination between human first responders and robotic systems, a prime example being a quadruped robot tailored for SAR tasks Sanfilippo and Rano (2023). The integration of hand gesture-based control into the functionality of a quadruped robot for SAR operations may signify a paradigm shift in the field of robotics. The canine-inspired robot, designed to navigate challenging terrains and aiding in rescue missions, aligns with the agility and adaptability of real-life SAR dogs. Leveraging hand gestures as a control mechanism for this robot not only bridges the communication gap between humans and machines but also ensures seamless and immediate commands, mirroring the fluidity of human-dog interaction. This innovation holds tremendous importance in the realm of SAR, as it enhances operator control, responsiveness, and precision, ultimately contributing to faster and more effective actions during critical operations. The fusion of advanced robotics and intuitive human-machine interfaces, represented by hand gesture-based control, promises to redefine the landscape of SAR robotics, potentially saving lives in the process.

### 1.1 Contributions and paper organisation

The key contributions of this work are.

• Introduced the innovative Massachusetts Institute of Technology (MIT) Quadruped Robot, controlled intuitively via hand gestures.
• Showcased the integration of a state-of-the-art Sparse Convolutional Neural Network—Bi-Long short term memory—gated recurrent unit network (SConv-Bi-LSTM-GRU) network, merging advanced machine learning with robotic systems.
• Implemented novel Meerkat optimisation for hyperparameter tuning, enhancing neural network performance and efficiency.
• Developed a cutting-edge hand gesture recognition system using computer vision, enabling real-time, accurate gesture interpretation for robot control.
• Utilised the YOLOv8 network on a custom Human Dataset for disaster scenarios, ensuring model adaptability and effectiveness in real-world search and rescue.
• Validated the methodology with experiments in simulated disaster scenarios, demonstrating the potential of deep learning and camera-based technologies in advancing UGV technology for disaster response.
• Advanced search and rescue robotics by proposing a holistic framework that integrates gesture-based UGV control with camera-based human detection, significantly improving operational outcomes and detection accuracy.

The remainder of the paper is organised as follows: Section 2 provides a detailed modelling of quadruped robot. Section 3 elaborates the proposed technique in detail. While Section 4 offers a detailed description of the dataset. Section 5 presents the results and discussion, and finally, Section 6 concludes this work.

## 2 Modelling of quadrupled robot

In this research, we examine the design of a resilient and agile quadruped robot, specifically focusing on the Massachusetts Institute of Technology (MIT) quadruped robot model. A concise overview of the robot’s modelling Bledt et al. (2018), is provided in this section.

An event-based finite state machine and an independent phase variable for each leg dictate the robot’s movement. This set-up is utilised to determine when each leg should be in contact with the ground or in a swinging motion. The system offers versatility in movement patterns, including trotting, bounding, pacing, and facilitating the addition of new patterns. These gaits are designed to mimic the movement of real cheetahs by regulating the phases of individual legs. In the event of unexpected leg contact events, adjustments are made to the nominal gait plan. Scheduled contacts are defined by independent Boolean variables *s*_*ϕ*_ ∈ {0 = *swing*, 1 = *contact*}, while estimated contacts are denoted by *s* ^∧^ ∈ {0 = *swing*, 1 = *contact*}. Using this data, the robot can distinguish between regular operation, unexpected and untimely contacts, and missed contacts delayed, subsequently modifying its control actions accordingly.

The quadrupedal controlling model integrates a linear correlation between the translational acceleration of the robot’s center of mass $\left(\ddot{q}c\right)$ and the angular acceleration of its body $\left(\dot{\omega }\beta \right)$ , concerning the forces $G={\left(G_{1}^{T},G_{2}^{T},G_{3}^{T},G_{4}^{T}\right)}^{T}$ acting on each of the robot’s four feet. The controlling model can be represented as:

$$\underbrace{\left[\begin{array}{ccc} X_{3} & \ldots & X_{3}\\ \left[q_{1}-q_{c}\right]\times & \cdots & \left[q_{4}-qc\right]\times \end{array}\right]}CG=\underbrace{\left[\begin{array}{c} m\left(\ddot{q}c+k\right)\\ YG\dot{\omega }\beta \end{array}\right]}d$$ (1)

where *m* and **Y**_*G*_ denote the overall mass and centroidal rotational inertia of the robot, respectively. Moreover, **k** represents the gravity vector, and **r**_*i*_ (for *i* ∈ 1, 2, 3, 4) are the positions of the robot’s feet. The expression $\left[r_{i}-q_{c}\right]\times$ refers to the skew-symmetric matrix representing the cross product $\left[r_{i}-q_{c}\right]\times G_{i}$ .

One of the operational modes for leg control in quadruped robot is the Balance Controller, inspired by a modified implementation of the control method outlined in Focchi et al. (2017). This controller applies proportional-derivative (PD) control to regulate the robot’s center of mass and body orientation while satisfying friction constraints on foot forces. The proportional-derivative (PD) control law is expressed in Eq. 2:

$$\left[\begin{array}{c} \ddot{q}c,\delta \\ \dot{\omega }b,\delta \end{array}\right]=\left[\begin{array}{c} Gp,q\left(qc,\delta -qc\right)+Gd,q\left(\dot{q}c,\delta -\dot{q}c\right)\\ Gp,\omega \log\left(R\delta R^{T}\right)+Gd,\omega \left(\omega b,\delta -\omega \right) \end{array}\right]$$ (2)

The intended angular acceleration follows PD control on SO(3), utilising rotation matrices **R**_*δ*_ and **R** to denote the desired and actual body orientations, respectively. The orientation error is calculated using the exponential map representation of rotations.

The primary objective of the Equilibrium Regulator is to enhance the allocation of leg forces L to steer the estimated dynamics of the center of mass toward the target dynamics, as specified by:

$$ed=\left[\begin{array}{c} m\left(\ddot{q}c,\delta +\gamma \right)\\ IM\dot{\omega }b,\delta \end{array}\right]$$ (3)

Since the model represented in Eq. 1 is linear, the controller’s operation can naturally be expressed as the solution of a quadratic program (QP) as shown in Eq. (4):

$$\begin{array}{ll} F^{*} & =\underset{F\in R^{12}}{\min}{\left(AF-b_{d}\right)}^{T}S\left(AF-b_{d}\right)+\alpha \|F{\|}^{2}\\ & \;+\beta {\left\|F-F_{\text{prev }}^{*}\right\|}^{2}\;\text{ s.t. }\;CF\leq d \end{array}$$ (4)

The cost function illustrated in Eq. (3) embodies an equilibrium among three primary objectives: steering the center-of-mass (CoM) dynamics towards the desired trajectory, reducing the applied forces, and penalising disparities between the present Quadratic Program (QP) outcome and the previous time step’s solution, denoted as $F_{\text{prev}}^{*}$ . The matrix **S** dictates the relative emphasis on managing rotational versus translational motion. Additionally, positive gains *α* and *β* dictate the influence of force standardisation and solution refinement. The constraints **CF** ≤ **d** are essential to ensure that the optimised forces adhere to the friction pyramid and that the normal forces fall within feasible limits. These constraints dynamically switch between the support-leg and swing-leg bounds based on the scheduled contact *s*_*ϕ*_, as elaborated earlier.

## 3 Proposed model for hand gesture detection

### 3.1 Convolutional neural network (CNN)

Convolutional Neural Networks (CNNs) are specialised deep learning models for processing data like images and signals. This text emphasises 1D CNNs, suited for one-dimensional data such as time series Ayadi et al. (2021). The fundamental operation in a 1D CNN is the convolution, involving a signal *X* and a filter *F*, defined by $\left(Y*F)(t)=\sum_{i=0}^{k-1}X(t+i)\cdot F(i\right)$ , where *Y* is the output. 1D max-pooling, another key operation, downsamples the output, enhancing feature detection by keeping the maximum value over a specified window.

### 3.2 Bidirectional LSTM model

**FIGURE 1:**
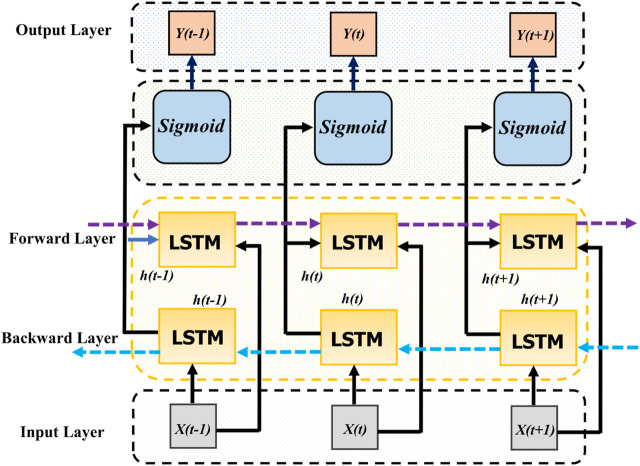
General structure of Bi-directional LSTM model.

Bi-LSTM, enhancing traditional RNNs, processes sequences in both forward and backward directions to better capture context Hameed and Garcia-Zapirain (2020). At its core, an LSTM cell has three gates controlling data flow and updates the unit’s state with inputs. Bi-LSTM integrates forward and backward LSTM layers, as shown in Figure 1, with outputs combined at each time step, enriching the sequence representation by incorporating information from both directions.

### 3.3 Gated recurrent unit (GRU)

**FIGURE 2:**
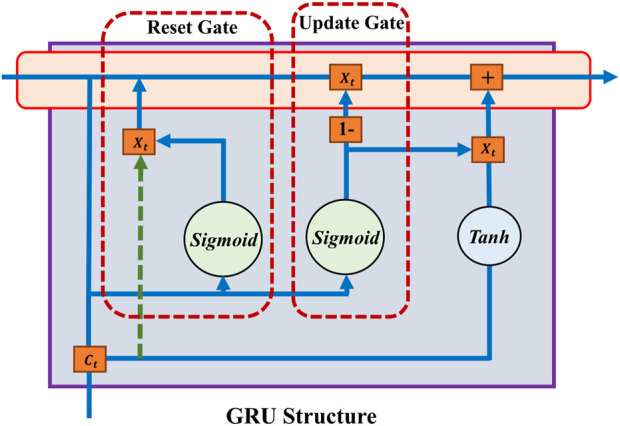
Gru module structure.

GRUs are a streamlined alternative to LSTMs designed to tackle the vanishing gradient problem in RNNs, facilitating long-range sequence learning Islam and Hossain (2021). A GRU has two gates: reset and update, as shown in Figure 2, simplifying the structure while maintaining the capacity to manage data flow. The unit’s state is updated through a blend of past state information and new inputs, guided by these gates, ensuring efficient dependency capturing in sequential data.

### 3.4 SConv-Bi-LSTM-GRU

The CNN-Bi-LSTM-GRU model represents an advanced architectural approach designed for the nuanced processing of sequential data, blending the strengths of convolutional neural networks (CNNs), bidirectional long- and short-term memory (Bi-LSTM) units, and gated recurring units (GRUs) into a singular, composite framework. This model stands out for its capability to adeptly handle a multitude of sequence-based challenges, benefiting from the synergistic integration of its components. Initially, the CNN layers take precedence in the model’s operation, tasked with the extraction of pivotal local features from the input data. This is notably effective even in scenarios involving one-dimensional sequence data, highlighting the CNN’s adaptability in identifying relevant spatial hierarchies and features within the sequence.

As the process advances, the Bi-LSTM layer comes into play, serving a critical role in discerning dependencies that span both backward and forward within the data sequence. This is achieved through the deployment of two distinct LSTM layers, each oriented in opposite directions (forward and backward), thereby ensuring a comprehensive analysis of temporal dynamics across the sequence. The merging of the forward and backward hidden states at every timestep enables the construction of a more complete and nuanced representation of the sequence, enhancing the model’s predictive accuracy.

Subsequent to the Bi-LSTM stage, the GRU component is introduced to further refine the output. GRUs are celebrated for their efficiency in modeling temporal dependencies within sequences, offering a streamlined alternative to traditional RNNs with a comparable capacity for capturing essential context and long-term dependencies. This characteristic makes GRUs an invaluable addition to the model, bolstering its ability to process and interpret sequential data with greater depth.

**FIGURE 3:**
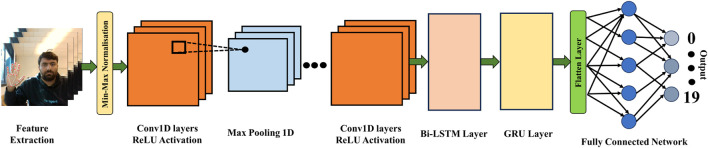
General architecture of the proposed model.

The architecture typically culminates with the integration of one or more fully connected layers, which serve to consolidate and interpret the processed data for a final outcome, The proposed structure of the model is shown in Figure 3. Through this comprehensive and meticulously designed structure, the CNN-Bi-LSTM-GRU model emerges as a highly versatile and powerful tool, finding application across a diverse array of fields such as natural language processing, speech recognition, and time series analysis. Its unique combination of feature extraction, forward and backward temporal dependency modelling, and context capture capabilities render it an exceptionally robust solution for the challenges inherent in sequential data processing tasks.

The recognition of hand gestures requires a comprehensive approach capable of understanding both spatial and temporal aspects of the data. The proposed CNN-Bi-LSTM-GRU architecture is uniquely suited to this task for the following reasons.

• **CNN for Spatial Feature Extraction:**
  • *Rationale:* Hand gestures contain complex spatial patterns that are crucial for differentiation. CNNs excel in extracting hierarchical spatial features from these images, capturing essential details like hand positions and orientations.
  • *Benefit:* This ensures the model accurately identifies key spatial features of hand gestures, enabling effective differentiation based on visual characteristics.
• **Bi-LSTM for Temporal Dependency Modelling:**
  • *Rationale:* Gestures are sequential in nature, requiring an understanding of both preceding and succeeding movements. Bi-LSTMs process data in both directions, capturing nuanced temporal patterns and dependencies.
  • *Benefit:* Incorporating Bi-LSTMs allows the model to recognise gestures based on the sequence of movements, enhancing prediction accuracy and robustness.
• **GRU for Efficient Sequence Modelling:**
  • *Rationale:* Efficient processing of temporal sequences is crucial for real-time gesture recognition. GRUs provide a simpler alternative to LSTMs for modelling temporal dependencies, maintaining performance while reducing computational complexity.
  • *Benefit:* GRUs ensure the architecture remains computationally efficient and capable of capturing essential dynamics of hand gesture sequences, suitable for real-time applications.

### 3.5 Hyperparameters of SConv-Bi-LSTM-GRU

The CNN-Bi-LSTM-GRU model involves a set of critical hyperparameters that significantly influence its performance. Starting with the convolutional layers (CNN), the number of layers typically ranges from one to five, where a deeper network may be advantageous for more intricate features but runs the risk of overfitting. Filter sizes and strides, which usually vary between one and five, determine the granularity of features the CNN captures, allowing the model to handle both fine-grained and broad patterns. In the Bidirectional LSTM (Bi-LSTM) component, the number of LSTM layers varies between one and three. A deeper architecture can effectively capture long-range dependencies, but it is computationally more intensive. Setting the number of hidden units, which ranges from 64 to 512, impacts the model’s capacity to learn complex relationships in the data. Additionally, selecting an appropriate dropout rate, typically between 0.2 and 0.5, plays a crucial role in mitigating overfitting. For the Gated Recurrent Units (GRU), you can configure the number of hidden units in a similar range (64–512) to control the model’s expressiveness. The dropout rate also applies here, with values between 0.2 and 0.5 serving as effective choices for regularisation.

Other crucial hyperparameters include the learning rate, which often falls within the range of 0.0001–0.01, the choice of optimiser (e.g., Adam or RMSprop), the selection of an appropriate loss function based on the specific task (e.g., cross-entropy for classification), and the batch size, typically ranging from 16 to 128. Epochs determine how many times the model iterates over the entire dataset, typically ranging from 10 to 100, while early stopping criteria depend on the problem and can involve parameters like patience (5–20) and improvement threshold (0.001–0.01). Finding the optimal combination of these hyperparameters often involves experimentation and hyperparameter tuning techniques, adjusting them within these ranges to maximise model performance while preventing overfitting or underfitting. The optimised hyperparameters are presented in Table 1.

**TABLE 1:** Hyperparameters of the proposed model.

Layer type	Hyperparameter	Value
Conv1D	Number of filters	64
Conv1D	Kernel size	2
Conv1D	Activation function	relu
Conv1D	Number of filters	32
Conv1D	Kernel size	2
Conv1D	Activation function	relu
MaxPooling1D	Pool size	2 (default)
Bidirectional LSTM	Units per direction	64
Bidirectional LSTM	Return sequences	True
GRU	Units	64
GRU	Return sequences	False
Dense	Units	20
Dense	Activation function	softmax
Model Compilation	Loss function	sparse_categorical_crossentropy
Model Compilation	Optimiser	adam
Model Compilation	Metrics	accuracy

### 3.6 Meerkat optimisation algorithm (MOA)

The Meerkat Optimisation Algorithm (MOA) draws inspiration from the survival and behaviour patterns of meerkats in their natural habitat Xian and Feng (2023). Meerkats exhibit remarkable traits, such as their acute sense of smell, which aids in food discovery, and their collaborative hunting efforts facilitated by purring sounds. Additionally, meerkats employ a sentinel system wherein some individuals monitor their surroundings from elevated vantage points, issuing warning calls when predators are detected, prompting quick group concealment. When faced with a predator, meerkats employ tactics like displaying their teeth and claws while lying on their backs to deter attacks, or they may stand together, arch their backs, raise fur, and hiss to appear as a formidable collective entity. These varying behavioural strategies, encompassing hunting, vigilant sentinels, fleeing, and confrontation, enable meerkats to effectively balance their foraging for sustenance and resource exploration with the constant vigilance required to detect and counteract threats in their challenging desert environment.

### 3.7 Initialisation

Generate an initial population *p* of *M* mongooses with *E* dimensions using Eq. 5:

$$P=\left[\begin{array}{c} \left[P_{1,1},P_{1,2},\ldots ,P_{1,k},\ldots ,P_{1,E}\right]\\ \left[P_{2,1},P_{2,2},\ldots ,P_{2,k},\ldots ,P_{2,E}\right]\\ \vdots \\ \left[P_{i,1},P_{i,2},\ldots ,P_{i,k},\ldots ,P_{i,E}\right]\\ \left[P_{M,1},P_{M,2},\ldots ,P_{M,k},\ldots ,P_{M,E}\right] \end{array}\right]$$ (5)

where *P*_*i*_ = [*P*_*i*,1_, *P*_*i*,2_, … , *P*_*i*,*k*_, … , *P*_*i*,*E*_] represents the i-th mongoose (candidate solution), M = the total number of mongooses (population size), *E* = the dimension of the problem, and k ranges from one to E, *P*_*i*,*k*_ = initialized using a random normal distribution within upper and lower bounds.

### 3.8 Hunting and vigilance

Save the initial position using Eq. 6:

$$direct=X_{0,i}$$ (6)

where *X*_0,*i*_ is the initial position of the *i-th* meerkat.

And to Calculate the step size using Eq. 7:

$$step=\left(1-\frac{t}{T}\right)\cdot r$$ (7)

where *t* is the current iteration, *T* is the maximum number of iterations, and *r* is a random number.

To update the meerkat’s position Eq. 8 is used:

$$X_{t+1,i}=X_{t,i}+step\cdot direct$$ (8)

where *X*_*t*+1,*i*_ is the new position of the *i-th* meerkat, *X*_*t*,*i*_ is the current position, and *direct* is the initial position.

Meerkats can also hunt together using Eq. 9

$$X_{t+1,i}=X_{t,i}+step\cdot \left(X_{t,j}-\left(rand+0.5\right)\cdot X_{t,i}\right)$$ (9)

where *X*_*t*,*j*_ is the position of a randomly selected *j-th* meerkat, and *rand* is a random number.

### 3.9 Fleeing or fighting enemies

The emergency position can be calculated using Eq. 10.

$$X_{t,emergency}=X_{t,i}+\left(2\cdot rand\cdot X_{t,gb}-X_{t,i}\right)$$ (10)

where *X*_*t*,*gb*_ is the best position found so far.

To Update the position based on fitness comparison, following Eq. 11 will be used.

$$\begin{array}{ll} Iff\left(X_{t,emergency}\right) & <f\left(X_{t,i}\right),thenX_{t+1,i}\\ & =X_{t,emergency};else,X_{t+1,i}\\ & =div\cdot X_{t,i}-\left(2\cdot rand\cdot X_{t,gb}-X_{t,i}\right) \end{array}$$ (11)

### 3.10 Random direction exploration

Levy flight is used for the exploration which is shown in Eq. 12:

$$X_{t+1,i}=X_{t,i}+\left(2\cdot rand-1\right)\cdot \left(X_{t,i}+rand\cdot s\right)\cdot step$$ (12)

where *s* is the Levy flight step size calculated using a Levy distribution.

### 3.11 MOA-SConv-bi-LSTM-GRU

**FIGURE 4:**
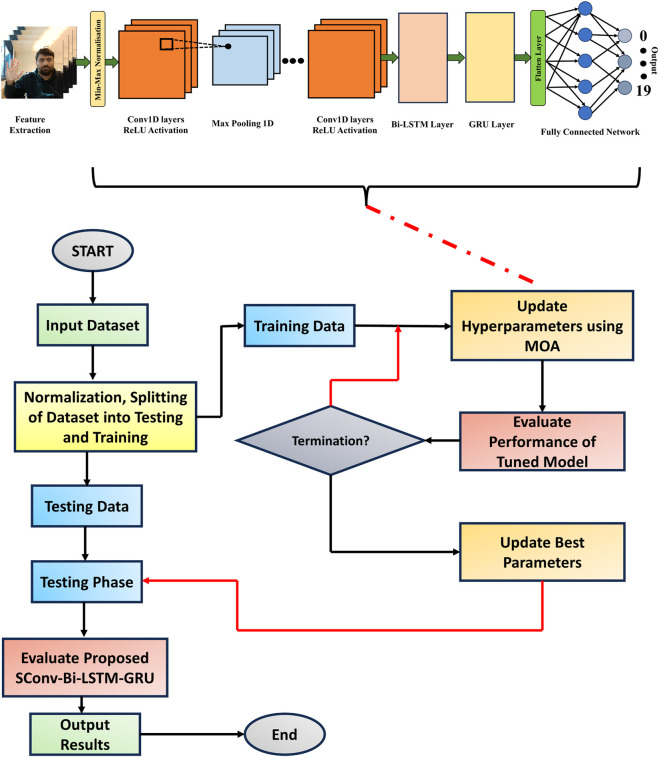
Flow Chart of the proposed MOA based Deep Neural Network Model.

In order to achieve optimal performance of the Conv-Bi-LSTM-GRU model on the hand gesture classification, we tuned the hyperparameters using the recently proposed Meerkat Optimisation Algorithm (MOA). MOA is a nature-inspired metaheuristic algorithm that simulates the foraging and sentinel behaviours of meerkats to balance exploration and exploitation in the search space. The hyperparameters tuned included number of convolutional filters, convolutional filter sizes, LSTM units, GRU units, dropout rate, batch size and learning rate. The flow of proposed MOA based deep neural network model is shown in Figure 4. The search ranges for these hyperparameters were set based on common practices. The objective function was validation accuracy of the Conv-Bi-LSTM-GRU model on a held-out set. MOA starts with an initial random population of solutions in the search space. The exploration phase consists of hunting behaviour, in which meerkats diffuse outwards from initial positions to forage, as well as coordinated hunting with other companions. The exploitation behaviour includes fighting against enemies by moving towards the historically best solution, or fleeing in the opposite direction. Additionally, random direction exploration facilitated by Lévy flights helps jump out of local optima. The sentinel mechanism balances exploration and exploitation by probabilistically switching between these behaviours. Over successive generations, MOA converges towards optimally performing hyperparameter configurations. We set the population size to 50 and maximum generations to 100 in our experiments. The optimal hyperparameter configuration discovered by MOA resulted in a test accuracy of greater than 98% on the gesture classification, which was superior to results from grid search and random search. The ability of MOA to balance local and global search, along with escaping local optima, made it well-suited for tuning the complex hyperparameter space of the Conv-Bi-LSTM-GRU model. The results validate the efficiency of the MOA algorithm for hyperparameter optimisation tasks.

### 3.12 *YOLOv8* architecture

The architecture of *YOLOv8* is detailed in Terven et al. (2023). It shares a structural resemblance to YOLOv5 but incorporates notable modifications to the CSPLayer, now referred to as the C2f module. The C2f module, which stands for cross-stage partial bottleneck with two convolutions, has been enhanced to integrate contextual information with high-level features to improve detection accuracy.

In *YOLOv8*, objectness, classification, and regression tasks are handled independently through an anchor-free model with a decoupled head. This design allows each branch to focus on its specific role, ultimately enhancing the overall model accuracy. The objectness score in the *YOLOv8* output layer is activated using the sigmoid function, indicating the likelihood of an object’s presence in the bounding box. For expressing the probability of objects belonging to each potential class, the softmax function is employed for class probabilities. The classification loss in *YOLOv8* is computed using binary cross-entropy, while bounding box loss utilises CIoU Zheng et al. (2020) and DFL Li et al. (2020). These loss functions contribute to improved object detection performance, particularly for smaller objects.

In addition to its object detection capabilities, *YOLOv8* introduces a semantic segmentation model known as *YOLOv8*-Seg. Unlike the conventional YOLO neck design, the C2f module follows the CSPDarknet53 feature extractor as the primary component. Two segmentation heads, trained to predict semantic segmentation masks for input images, follow the C2f module. Featuring five detection modules and a prediction layer, the detection heads of the *YOLOv8*-Seg model are comparable to those of *YOLOv8*. The *YOLOv8*-Seg model has demonstrated state-of-the-art results in various object identification and semantic segmentation benchmarks, maintaining high speed and efficiency.

## 4 Dataset description of hand gesture recognition

### 4.1 Hand landmarks extraction

Efficient landmark extraction utilises the Mediapipe pose detector, an open-source cross-platform tool that leverages machine learning algorithms to track hands in colour images Zhang et al. (2020). This detector excels in accurately localising hand landmarks across diverse pose configurations, employing a two-step process involving a palm detector followed by a hand model to ascertain 2D positions of 21 hand joints. This methodology minimises the need for data augmentation, addressing challenges related to rotation, translation, and scale, thus prioritising localisation accuracy. Moreover, the approach optimises the detection process by leveraging prior landmark predictions, enabling real-time detection of multiple hands. The dataset comprises 16,000 samples from four individuals, each contributing 200 samples for 20 distinct gestures, split into 80% training and 20% testing sets. The corresponding robot actions for each gesture are outlined in Table 2.

**TABLE 2:** Gestures explanation to control the selected robot.

Gestures	Action	Summary of action
1	Start	Robot will start and wait for commands
2	Stop	Robot will stop
3	Move forward	Robot will move forward
4	Move reverse	Robot will move backward
5	Move left	Robot will move left
6	Move right	Robot will move right
7	Move Top Left	Robot will Move to Top Left
8	Move Top Right	Robot will Move to Top Right
9	Move Bottom Left	Robot will Move to Bottom Left
10	Move Bottom Right	Robot will Move to Bottom Right
11	Jump Up	Robot will Jump Upwards
12	Increase Max Speed	Increase max speed by 10%
13	Decrease Max Speed	Decrease max speed by 10%
14	Increase Linear Speed	Increase linear speed by 10%
15	Decrease Linear Speed	Decrease linear speed by 10%
16	Increase Angular Speed	Increase angular speed by 10%
17	Decrease Angular Speed	Decrease angular speed by 10%
18	Pause	Pause at current position
19	Move Down	Robot will move down
20	Holmonic Mode	It will Enable Holmonic Mode
No hand	Stop	Robot will stop

### 4.2 Dataset pre-processing

Normalisation is a crucial pre-processing step in data analysis and machine learning, aimed at standardising the scale of features within a dataset. The min-max normalisation technique is one such approach that scales the features to a specific range, (0, 1), making the data comparable and aiding in better convergence during training.

The Min-Max Normalisation is calculated using the Eq. 13 for each feature *x* in the dataset:

$$x_{\text{normalised}}=\frac{x-\text{min}\left(x\right)}{\text{max}\left(x\right)-\text{min}\left(x\right)},$$ (13)

where min(*x*) is the minimum value of the feature *x* and max(*x*) is the maximum value of the feature *x* in the dataset.

By employing min-max normalisation, we transform the features to fall within the (0, 1) range, preserving the relationships and distributions within the data. This aids in the effective use of machine learning algorithms that are sensitive to feature scales.

### 4.3 Dataset analysis

**FIGURE 5:**
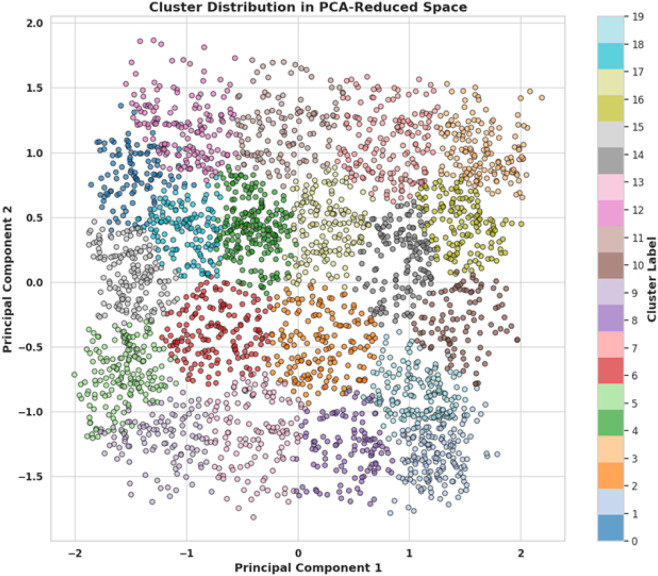
Distribution of clusters in a two-dimensional space defined by the first two principal components.

The scatter plot, shown in Figure 5, illustrates the distribution of clusters in a two-dimensional space defined by the first two principal components derived from a PCA transformation. Each point represents an individual data sample, colour-coded to indicate cluster membership, with a total of 20 distinct clusters as shown by the colour bar on the right. The distribution of colours across the plot signifies the degree to which the PCA has managed to separate different clusters in the reduced-dimensional space. Clusters are spread across the plane of the first two principal components, with varying degrees of overlap. Some clusters, particularly those in the center of the plot, appear to intermingle, suggesting that the separation between these groups in the original high-dimensional space is not as pronounced. In contrast, some clusters, especially those on the periphery, exhibit more distinct groupings, indicating a greater separation from others. This visualisation provides a compelling overview of the data structure and the effectiveness of PCA in reducing dimensionality while maintaining the inherent clustering of the dataset. The clear differentiation of some clusters and the overlap of others may indicate potential patterns or relationships within the data that merit further investigation. It also serves as a visual assessment of the cluster model’s performance, showing how well the clusters identified by an algorithm like K-Means correspond to the reduced dimensionality representation provided by PCA. The plot is well-suited for a research setting, offering both a high-level overview of data segmentation and a starting point for more detailed statistical analysis.

**FIGURE 6:**
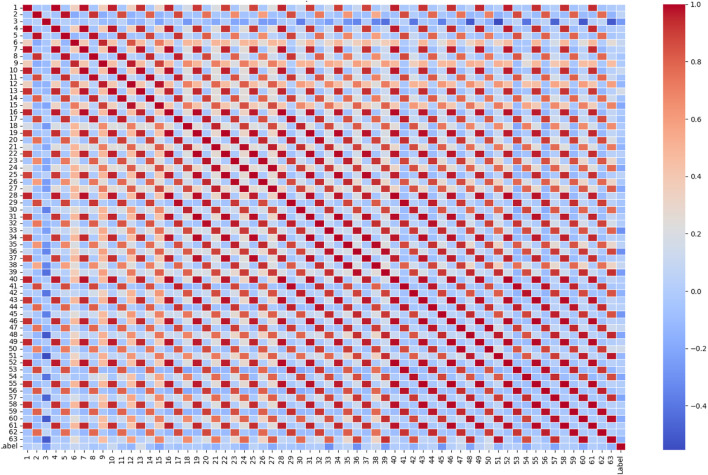
Correlation matrix heatmap for the features along with the target variable of a dataset.

Figure 6 showcases a correlation matrix heatmap for the top five features along with the target variable of a dataset. The shades of red and blue across the heatmap represent the strength and direction of the correlation between each pair of features. A correlation value of 1.00 along the diagonal confirms that each feature perfectly correlates with itself, as expected. The colour intensity and the correlation values indicate the relationship strength; deep reds signify a strong positive correlation, blues indicate a negative correlation, and lighter colours correspond to weaker correlations. Observing the correlations between the features and the target, “Feature 2” shows a negative correlation with the target variable, indicated by a light blue shade and a value of approximately −0.26. This implies that as “Feature 2” increases, the target variable tends to decrease, albeit not very strongly. Other features, such as “Feature 62,” “Feature 50”, “Feature 53,” and “Feature 56,” display varying degrees of positive intercorrelations, evidenced by the predominantly red off-diagonal blocks, some of which are very strong (close to 1), suggesting that these features may share a significant amount of information or could be redundant. Interestingly, these features do not exhibit strong correlations with the target, as seen by the more subdued colours in the target row/column, indicating that while they are strongly related to each other, they may not individually influence the target variable significantly. The heatmap is a powerful tool for quickly visualising the presence and intensity of relationships between variables and is essential for identifying potential features for model input, assessing multicollinearity, and directing further data analysis efforts. The chart’s clear labelling and distinct colour coding make it an effective visual summary for both data exploration and presentation in a research context.

### 4.4 Description of human detection dataset

A new simulated disaster victim image and video dataset was recently created under ethical conditions Dadwhal et al. (2019). Volunteers safely participated with due care for their wellbeing. Fuller’s earth, a skin-safe compound, simulated disaster scene dust on the volunteers posing as victims in different positions amidst realistic rubble and clutter. The objective was capturing images of humans in poses as might be found after a catastrophic event, with the volunteers including one woman and four men. The scene includes variations in colour, scale, pose, illumination, motion blur and occlusion across 128 still images and 15 video clips. The dataset has pixel-level annotation of skin regions to enable developing and testing algorithms to assist first responders in disaster victim location and rescue. Unique in providing victim simulation data, it serves as a benchmark for research toward automated disaster victim detection and location in cluttered scenes.

The Human Detection dataset, comprising 6,447 images from disaster scenarios, underwent a preprocessing step to align with the specifications of *YOLOv8*. As part of this preprocessing, the images were resized to fulfil the specific requirements of the *YOLOv8* model. The resizing process was carefully executed to maintain the integrity of the data while ensuring compatibility with the *YOLOv8* architecture. Following this adjustment, the dataset was further annotated using the professional online tool ROBOFLOW, with a meticulous focus on selecting human instances and accurately delineating them with bounding boxes. Subsequently, the resized and annotated dataset was strategically partitioned into training (70%), validation (15%), and testing (15%) subsets to facilitate effective model training and rigorous evaluation.

## 5 Results and discussion

### 5.1 Experimental setup

**FIGURE 7:**
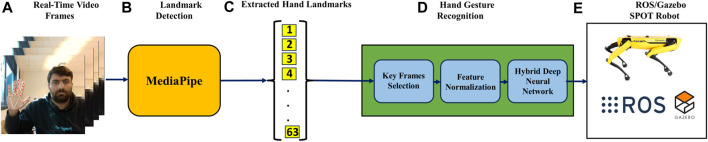
Proposed structure of the hand gesture recognition-based control of spot robot in ROS/Gazebo. **(A)** Real Time Video Frames, **(B)** Mediapipe Detects the hand land marks, **(C)** Land Marks are Exctracted, **(D)** Hand Gesture Recognition System using Deep Learning Model, and **(E)** Integration with ROS for Control.

**FIGURE 8:**
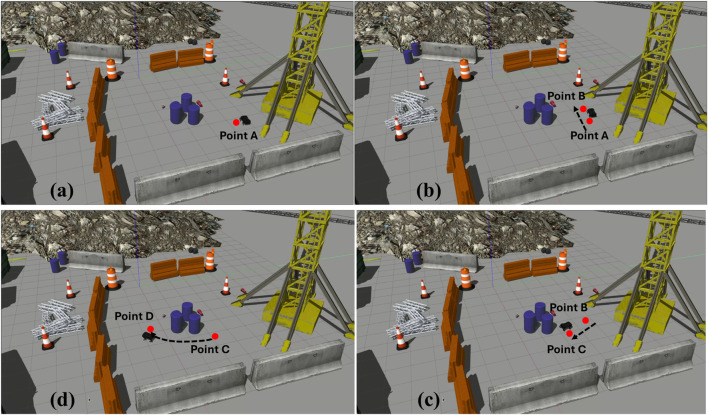
Sequence of Figures showcasing the movement of the quadruped robot according to the hand gestures in a dynamic close to real world environment **(A)** Quadruped Robot starts from Point A **(B)** By detecting the gesture “move backward,” the quadruped robot starts moving backward till Point B **(C)** Then the quadruped robot takes a right turn and moves forward to reach the Point C **(D)** After that the quadruped robot moves from Point C to Point D.

The experimental setup utilised a laptop powered by an AMD Ryzen 5 5500U processor with Radeon Graphics, operating at a base frequency of 2.10 GHz. The hardware facilitated the implementation of deep learning models and real-time processing for hand gesture detection. An Intel RealSense camera was employed for capturing image data and depth information crucial for accurate hand gesture analysis. The ROS/Gazebo based simulation of hand gesture control of quadruped robot is shown in Figure 8. The proposed real-time hand gesture-based control of the quadruple robot is implemented in the Robot Operating System (ROS) and Gazebo simulator Takaya et al. (2016), as shown in Figure 7.

### 5.2 Hand gesture recognition

#### 5.2.1 Comparative analysis

**FIGURE 9:**
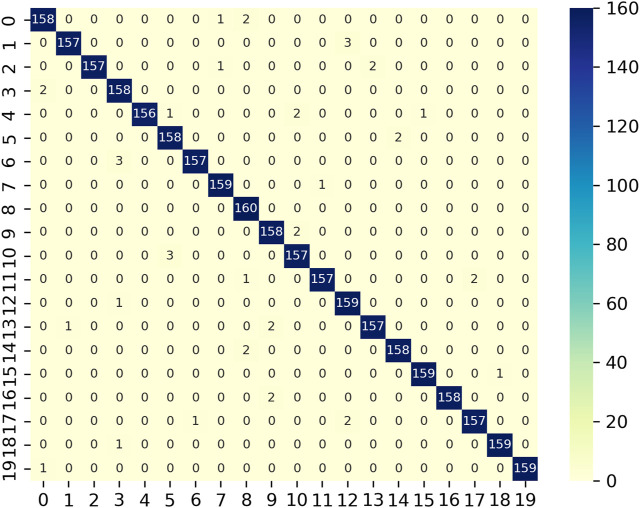
Confusion matrix for hand gesture classification of MOA-SConv-Bi-LSTM-GRU.

Hyperparameter optimisation is crucial for configuring the complex neural topology and training variables of deep learning architectures such as Conv-Bi-LSTM-GRU to achieve maximum effectiveness. We research the suitability of the novel Meerkat Optimisation Algorithm (MOA) for this problem against prevalent alternatives including Arithmetic Optimisation (AOA), Whale Optimisation (WOA), Grey Wolf Optimisation (GWO) and Particle Swarm Optimisation (PSO). The comparative evaluation on a hand gesture classification dataset reveals MOA’s outstanding performance. The confusion matrix of proposed technique is shown in Figure 9. The MOA-tuned Conv-Bi-LSTM-GRU model attains best-in-class testing accuracy of 98.66%, significantly outperforming runner-up AOA’s 97.25%. This difference is statistically significant as validated by hypothesis testing, demonstrating MOA’s superior search capabilities.

Recall is a crucial metric for text analytics measuring the model’s ability to correctly identify relevant documents based on the learned representation. The MOA-optimised configuration retrieves 98.66% related samples, compared to 97.11% by AOA-Conv-Bi-LSTM-GRU. The top-2 precision scores of 98.67% and 97.27% respectively highlight the high relevancy of documents flagged positive. The harmonic F1-score mean aggregates precision and recall performance. MOA surpasses other techniques by an F1 measure of 98.66%, reflecting its well-balanced tuning. AOA comes closest at 97.09% F1-value trailed by WOA, GWO and PSO in that order. Evidently, MOA discounts suboptimal settings best. In a nutshell, MOA’s exploration-exploitation equilibrium facilitated by the novel sentinel vigilance alongside hunting, combat and flight behaviours manifests salient optimisation capabilities outshining existing algorithms. It discovers close to optimal hyperparameter configurations to boost Conv-Bi-LSTM-GRU’s text mining efficacy. Our evaluations confirm that the unconventional meerkat-inspired MOA paradigm offers a valuable addition to the hyperparameter optimisation repertoire. MOA has also evidenced versatile adaptation across manifold problem domains. These results motivate harnessing MOA to optimise frontline deep neural paradigms for enhanced analytics. The detailed comparative analysis is presented in Table 3.

**TABLE 3:** Comparative analysis of the evaluation metrics achieved by competing techniques.

Technique	Accuracy	Precision	Recall	F1-score
MOA-SConv-Bi-LSTM-GRU	0.9866	0.9867	0.9866	0.9866
AOA-SConv-Bi-LSTM-GRU	0.9725	0.9727	0.9711	0.9709
WOA-SConv-Bi-LSTM-GRU	0.9463	0.9441	0.9425	0.9487
GWO-SConv-Bi-LSTM-GRU	0.9254	0.9228	0.9281	0.9297
PSO-SConv-Bi-LSTM-GRU	0.8813	0.8842	0.8867	0.8889

### 5.3 Victim detection

**FIGURE 10:**
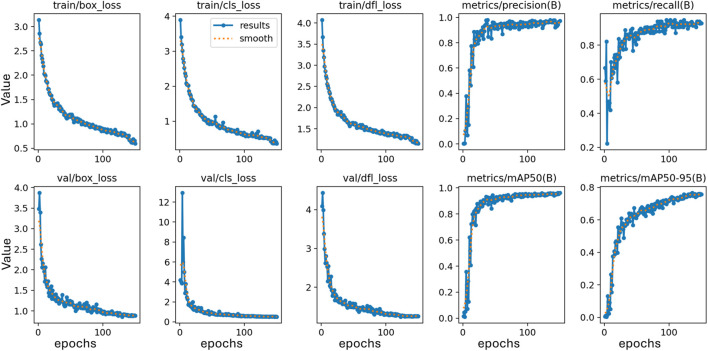
Training matrices variation over the iteration on Disaster Scenario Dataset.

**FIGURE 11:**
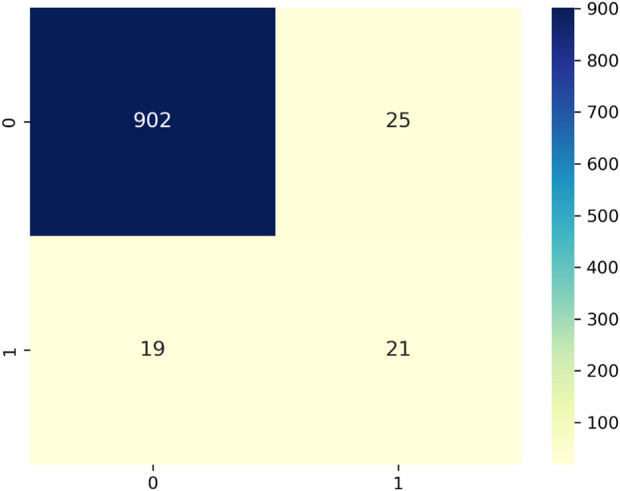
Confusion matrix for human detection in disaster scenario test data.

**FIGURE 12:**
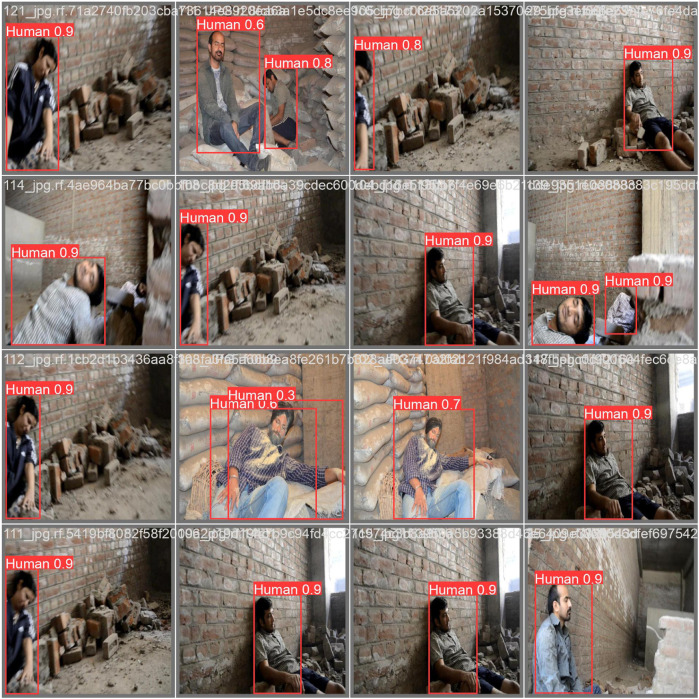
Prediction results for human detection on test data. Reprinted/adapted with permission from “Simulated Disaster Victim (SDV1 & SDV2) dataset” by Dadwhal et al. 2019, licensed under CC BY 4.0 DEED.

The training effectiveness of the model can be evaluated by examining the convergence of key parameters on the training and test data sets, as shown in Figure 10. This includes the box localisation loss, classification loss, objectness loss, mean Average Precision at different IoU thresholds, recall and precision. Lower loss values demonstrate better training, with the training and validation losses expected to steadily decrease then stabilise over epochs as the model fits the data. The results validate lack of overfitting, as the losses steadily drop till 200 epochs without spikes or oscillations. Precision, recall and mean Average Precision values are also examined over epochs, with regular patterns again confirming appropriate model convergence. Assessment of these metrics over the number of training iterations thus facilitates model selection, and hyperparameter tuning, and demonstrates the approach effectively learns to locate and classify the objects of interest without simply memorising the training examples. The confusion matrix for the proposed technique on testing data is shown in Figure 11. The victim detection on test dataset is shown in Figure 12.

Recognising the importance of addressing environmental and internal uncertainties in such operations is crucial. In Tutsoy (2022), the authors detail an AI-based, multi-dimensional policy making algorithm aimed at minimising casualties during pandemic diseases by incorporating both pharmacological and non-pharmacological strategies. Within the framework of this study, environmental uncertainties include unpredictable disaster conditions, such as terrain variability, weather changes, and debris presence, which significantly impact UGV navigation and victim identification. Internally, uncertainties stem from the limitations of the UGVs’ hardware and software, including sensor inaccuracies and the potential for computational errors in real-time processing. A integrated framework leveraging a deep learning model is developed in this study to facilitate intuitive, real-time control over UGVs. This allows for precise manoeuvrability in complex environments, addressing internal uncertainties by enabling operators to make split-second decisions based on live feedback from the field. Furthermore, the usage of YoLoV8, trained on specialised dataset, enhances the UGVs’ ability to accurately detect human victims under diverse and challenging conditions.

## 6 Conclusion

This paper introduces a pioneering and holistic approach to revolutionise search and rescue (SAR) operations in disaster environments through the integration of cutting-edge technologies into unmanned ground vehicles (UGVs). The proposed methodology, consisting of gesture-controlled UGV operation and camera-based human detection, addresses critical challenges in disaster response scenarios. The gesture-controlled UGV operation provides an intuitive interface for human operators, enabling precise control in confined and intricate spaces. The selected prime example is a quadruped robot tailored for SAR tasks. Leveraging a deep learning (DL) model, hand gestures are accurately interpreted and translated into real-time control commands, significantly enhancing situational awareness and control precision. The second component focuses on camera-based human detection, employing the *YOLOv8* DL network. This innovative approach effectively identifies individuals amidst disaster-induced debris and chaotic surroundings. The DL model is trained and tested using a specialised Human Dataset for disaster scenarios, ensuring its adaptability and efficacy in real-world search and rescue operations. Experimental results conducted in simulated disaster scenarios validate the proposed methodology’s efficacy and real-world viability. The integration of these components forms a cohesive framework that advances search and rescue capabilities, contributing to ongoing efforts to improve operational outcomes and ultimately save lives in disaster-stricken areas. This research underscores the potential of advanced technologies, including DL and camera-based approaches, in evolving UGV technology for disaster response. By addressing the challenges associated with confined spaces and human detection in chaotic environments, the proposed methodology represents a significant leap forward in the application of technology to enhance disaster response efforts. As technology continues to advance, the findings of this research contribute valuable insights to the ongoing quest for more effective and efficient SAR operations in the face of natural or man-made disasters. For future work, we aim to bridge the current gap by merging the gesture-based control of UGVs with advanced camera detection technologies for victim identification, setting the stage for a unified system that significantly enhances SAR capabilities.

## Data Availability

The original contributions presented in the study are included in the article/supplementary material, further inquiries can be directed to the corresponding author.
